# Pregnancy in a Patient With Cancer and Heart Failure: Challenges and Complexities

**DOI:** 10.6004/jadpro.2012.3.2.3

**Published:** 2012-03-01

**Authors:** Anecita P. Fadol, Tara Lech, Courtney Bickford, Syed Wamique Yusuf

**Affiliations:** From MD Anderson Cancer Center, Houston, Texas

## Abstract

A 24-year-old African American female (L.R.) with a history of smoking and gestational diabetes was diagnosed with Hodgkin lymphoma. She received multiple chemotherapies, including six cycles of ABVD (doxorubicin, bleomycin, vinblastine, and dacarbazine), followed by radiation therapy to left inguinal areas for a total of 30.6 Gy in 17 fractions; she obtained complete remission. Two years later, L.R. had disease relapse in the mediastinum and received two cycles of ESHAP (etoposide, methylprednisolone, high-dose cytarabine, cisplatin) followed by etoposide and ifosfamide. She then received BEAM (carmustine, etoposide, cytarabine, and melphalan) as a conditioning regimen and underwent autologous bone marrow transplant. Her post-transplant course was complicated by cytomegalovirus antigenemia, aspergillus pneumonia, and congestive heart failure with left ventricular ejection fraction (LVEF) of 20%–25%. She was treated with an ACE inhibitor (lisinopril) and a beta-blocker (carvedilol) with improvement of her LVEF to 30%–35%. A follow-up chest x-ray showed an increase in the size of the anterior mediastinal adenopathy suspicious for relapse of lymphoma, and at the same time she was also found to be 5 weeks pregnant.

Given her cardiomyopathy, significant obesity, poorly controlled diabetes, and cancer recurrence, L.R. was advised by her gynecologist that the pregnancy was very high risk and might not be viable. The oncologists advised her to terminate the pregnancy within the first trimester, as she needed salvage radiotherapy treatment to the mediastinum and chemotherapy treatments that might endanger the fetus. However, the patient decided to continue with the pregnancy. A multidisciplinary team—which included a cardiologist, oncologist, high-risk obstetrician, pharmacist, and nurse practitioner—was then involved to provide care during the pregnancy. A social worker was also solicited to help with home and financial issues because L.R. was a single mother with a 2-year-old son.

L.R. was treated with carvedilol and furosemide, with monthly cardiology clinical follow-up during the first and second trimesters, then every 2 weeks starting with the 28th week, and weekly thereafter until delivery. Between visits, she notified the clinic for symptoms of heart failure exacerbation and was seen as necessary. The possible in utero effects of both medications were discussed with the patient. L.R. had a normal uncomplicated pregnancy and delivered a 6-pound, 10-ounce healthy boy at 39 weeks via vaginal delivery and was discharged home 2 days later.

A week after delivery, L.R. presented to the cardiology clinic in good spirits and was excited to show pictures of her newborn baby. She had no cardiac complaints and the repeat echocardiogram showed an unchanged LVEF of 30%–35%.

The advent of newer treatment modalities has led to an increasing number of cancer survivors, and the number of women who have received cancer therapy with potential cardiotoxic side effects is growing rapidly. As these women contemplate pregnancy, history of prior cancer therapies is critical in determining the risk of cardiac complications during pregnancy. Cardiomyopathy is an adverse effect of many chemotherapeutic agents (Yeh & Bickford, 2009). Chemotherapy-induced cardiomyopathy may manifest before and during pregnancy and poses complex therapeutic challenges as medications such as angiotensin-converting enzyme (ACE) inhibitors are contraindicated in pregnancy because of their teratogenic effects (Briggs, Freeman, & Yaffe, 2008).

There is a paucity of information to guide the clinician in the management of these high-risk patients, who need meticulous surveillance and follow-up throughout the course of the pregnancy. The purpose of this article is to describe the collaboration of a multidisciplinary team of health-care providers in the management of a successful pregnancy in a cancer patient with heart failure (HF).

## Chemotherapy and Cardiotoxicity

Several of the standard chemotherapy regimens recommended for the treatment of Hodgkin lymphoma are anthracycline-based. In clinical trials, anthracyclines have proven to be highly efficacious in the treatment of lymphoma. Their efficacy has been attributed to a clear dose-response relationship, with higher doses showing greater rates of remission and cure (Shan, Lincoff, & Young, 1996). However, higher cumulative anthracycline doses are associated with an increased incidence of adverse effects, such as cardiotoxicity, which often limits the further use of certain cancer therapies.

Anthracyline-induced cardiotoxicity may be categorized into three distinct types: acute, early-onset chronic progressive, and late-onset chronic progressive (Grenier & Lipshultz, 1998; Lipshultz, Alvarez, & Scully, 2008; Yeh & Bickford, 2009). Acute cardiotoxicity occurs in < 1% of patients immediately after infusion of the anthracycline and may manifest as arrhythmias, acute pericarditis-myocarditis syndrome, or an acute, transient decline in myocardial contractility, which is usually reversible (Shan, Lincoff, & Young, 1996; Wouters, Kremer, Miller, Herman, & Lipschultz, 2005).

The early-onset chronic progressive form occurs in 1.6% to 2.1% of patients, during therapy or within the first year after treatment (Wouters et al., 2005; Yeh & Bickford, 2009). In a series of approximately 3,900 patients who received treatment with anthracyclines, heart failure occurred 0 to 231 days after the completion of anthracycline therapy (Von Hoff et al., 1979).

In contrast, late-onset anthracycline-induced cardiac abnormalities have been reported to occur much later, and may not become clinically evident until 10 to 20 years after the first dose of cancer treatment (Yeh & Bickford, 2009). Late-onset chronic progressive anthracycline-induced cardiotoxicity, which typically presents as dilated cardiomyopathy and can be progressive, occurs at least 1 year after completion of therapy in 1.6% to 5% of patients (Wouters et al., 2005; Yeh & Bickford, 2009).

Cardiotoxicity associated with anthracyclines is related to total cumulative dose. Studies that have looked at the cumulative probability of doxorubicin-induced heart failure have found that it occurs in 3% to 48% at doses of ranging from 400 to 700 mg/m2 (Von Hoff et al., 1979; Swain et al., 1997; Wouters et al., 2005). However, in a retrospective review of three trials, the incidence of HF was found to be 26% with cumulative doses of 550 mg/m^2^ (Swain, Whaley, & Ewer, 2003). Therefore, the maximum lifetime cumulative dose for doxorubicin is 400 to 550 mg/m^2^ (Wouters et al., 2005). In this case report, our patient received six cycles of ABVD that resulted in a total cumulative doxorubicin dose of 300 mg/m^2^.

Besides total cumulative dose, risk factors for anthracycline toxicity include IV bolus administration; higher single doses; history of prior irradiation; use of other concomitant agents known to have cardiotoxic effects, such as cyclophosphamide, trastuzumab (Herceptin), and paclitaxel; female gender; underlying cardiovascular disease; age (both young and old); and increased length of time since anthracycline completion (Grenier & Lipshultz, 1998; Swain, Whaley, & Ewer, 2003; Lipshultz, Alvarez, & Scully, 2008; Yeh & Bickford, 2009).

Although the cause of anthracycline-induced cardiotoxicity is probably multifactorial, free radical formation is generally acknowledged as the main mechanism (Yeh & Bickford, 2009). The semiquinone moiety of the doxorubicin increases oxygen radical activity thereby causing lipid peroxidation and cell injury (Shan, Lincoff, & Young, 1996). Other hypotheses include interference with topoisomerase II beta (Lyu et al., 2007; Yeh & Bickford, 2009), myocyte damage from calcium overload, adrenergic dysfunction, apoptosis, transcriptional changes in intracellular ATP production in cardiac myocytes, downregulation of mRNA expression for sarcoplasmic reticulum calcium-ATPase, which decreases cardiac contractility, and prolonged drug-related depression in cardiac glutathione peroxidase activity associated with mitochondrial DNA damage (Shan, Lincoff, & Young, 1996; Wouters et al., 2005; Yeh & Bickford, 2009).

## Management of Pregnancy With Heart Failure and Cancer

The management of pregnancy in the concurrence of heart failure and cancer presents an enormous challenge to health-care providers. The physiologic changes that occur in a normal pregnancy are stressful to the cardiovascular system. During pregnancy, blood volume increases 45% to 50% and cardiac output rises 30% to 50% above baseline, peaking by the end of the second trimester and reaching a plateau until delivery (Howlett et al., 2010; Rimes, Gano, & Milbourne, 2008).

Cardiac disease complicates approximately 1% to 3% of pregnancies, with a maternal mortality rate of 10% to 15% (Gei & Hankins, 2001; Klein & Galan, 2004). The risk of maternal death is approximately 7% if the patient is in New York Heart Association (NYHA) functional class III or IV (Thorne, 2004). An LVEF of < 20%, presence of mitral regurgitation, right ventricular failure, atrial fibrillation, and systemic hypotension are other risk factors that can increase the risk for overt heart failure during pregnancy and may increase the risk of maternal death (Thorne, 2004).

Patients with preexisting chemotherapy-induced left ventricular dysfunction during pregnancy should be closely monitored during the gestation period, at the time of delivery, and during the postpartum period (Howlett et al., 2010). Initial assessment should include a detailed history including cancer treatment history (chemotherapy and radiation therapy), review of systems including symptom assessment (i.e., orthopnea, paroxysmal nocturnal dyspnea, and weight gain), functional status, and baseline diagnostic tests (Table 1), which should be monitored closely on a regular basis (Howlett et al., 2010).

**Table 1:**
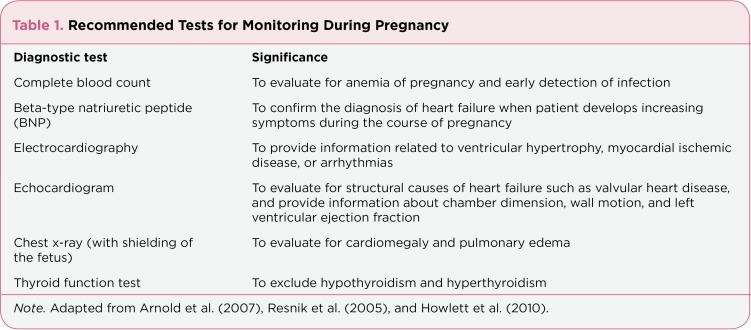
Table 1. Recommended Tests for Monitoring During Pregnancy

The hemodynamic changes that occur during pregnancy result in the development of several signs and symptoms that can mimic heart disease presentation. The normal symptoms of pregnancy can obscure the early signs and symptoms of heart failure exacerbation (Howlett et al., 2010). Manifestations of worsening heart failure that should prompt further investigation include chest pain, new-onset cough with dyspnea, increased jugular venous pressure/distention, new-onset diastolic murmur or systolic murmur (not considered physiologic), paroxysmal nocturnal dyspnea, pulmonary crackles or other adventitious breath sounds, and profound peripheral edema.

Early identification and intervention is crucial in preventing the worsening of heart failure. Because no specific criteria are available to differentiate subtle symptoms of heart failure from the normal course of pregnancy, health-care providers should maintain a high index of suspicion (Pearson et al., 2000). Careful and meticulous clinical assessment is necessary to distinguish the symptoms related to pregnancy vs. early-onset heart failure (Table 2).

**Table 2:**
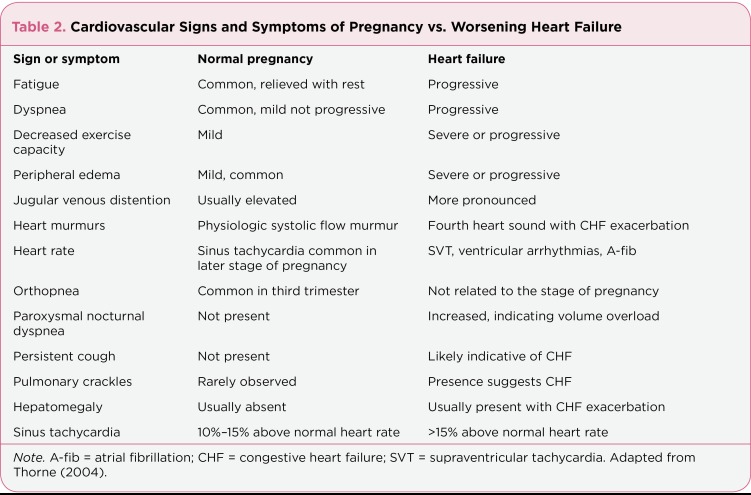
Table 2. Cardiovascular Signs and Symptoms of Pregnancy vs. Worsening Heart Failure

The goals of management and medical therapy are generally similar to those in the general population with heart failure, but with careful consideration of the impact of pharmacologic therapy on the fetus.

## Pharmacologic Management

The management of patients with heart failure is extremely complex; drug therapy must be tailored to fit the individual. There are many factors to consider when developing an adequate pharmacotherapeutic treatment plan, and decision-making can become increasingly more difficult during pregnancy. Patients ideally would remain on chronic therapies that have been shown to improve outcomes in heart failure; however, we must first evaluate the risk-benefit ratio of continuing each medication to minimize potential harm to the fetus. Table 3 highlights the medications currently prescribed for the treatment of heart failure and their associated risks to the unborn fetus. The following sections will take a closer look at each medication and review its evidence for use during pregnancy.

**Table 3:**
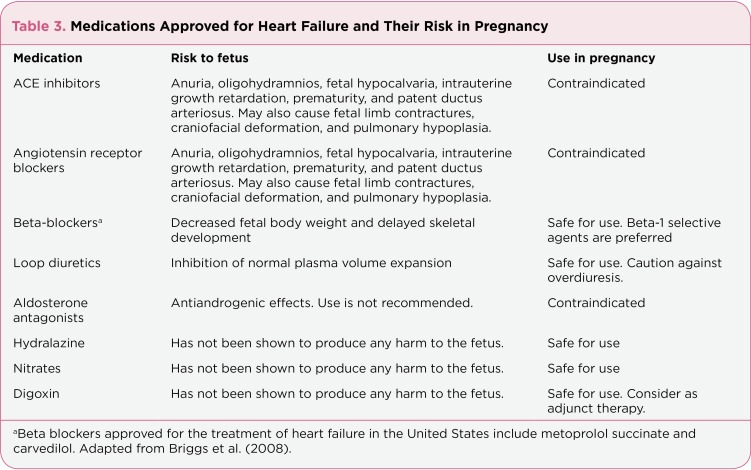
Table 3. Medications Approved for Heart Failure and Their Risk in Pregnancy

## ACE Inhibitors and ARBs

The use of ACE inhibitors and angiotensin receptor blockers (ARBs) is well established in the treatment of heart failure in nonpregnant patients (The SOLVD Investigators, 1991; Granger et al., 2003). These drugs inhibit the renin-angiotensin aldosterone system, and as a result they decrease blood pressure, reduce afterload, and improve LV systolic function. ACE inhibitors have been shown to improve morbidity, mortality, and quality of life in many large-scale prospective clinical trials (The SOLVD Investigators, 1991; The CONSENSUS Trial Study Group, 1987). ARBs are generally recommended as an alternative to ACE inhibitors in cases where ACE inhibitors are not tolerated (Granger et al., 2003). Treatment with either therapy, however, must be discontinued during pregnancy. Both ACE inhibitors and ARBs are known teratogens, and their use during pregnancy is contraindicated (Briggs, Freeman, & Yaffe, 2008).

Until recently it was thought that the risks associated with these drugs—including anuria, oligohydramnios, fetal hypocalvaria (reduced size of the calvarial bones), intrauterine growth retardation, prematurity, and patent ductus arteriosus—were highest during the second and third trimesters, but recent studies have reported adverse fetal outcomes associated with first trimester use as well (Lavoratti et al., 1997; Flather et al., 2000; Alwan, Polifka, & Friedman, 2005; Briggs, Freeman, & Yaffe, 2008; Buhimschi & Weiner, 2009). Cooper and colleagues (2006) reported that 7.1% of infants exposed to ACE inhibitors in the first trimester had congenital malformation, which was 2.71 times higher than the infants with no exposure (risk ratio, 2.71; 95% confidence interval, 1.72 to 4.27). Given the significant fetal risks, ACE inhibitors and ARBs should not be used during pregnancy.

## Beta-Blockers

Beta-blockers, such as metoprolol succinate and carvedilol, play an important role in the management of chronic heart failure patients. Beta-blockers reduced all-cause mortality and length of hospitalization in patients with heart failure (The MERIT-HF Study Group, 1999; Packer et al., 1996; Brophy, Joseph, & Rouleau, 2001). However, there is insufficient evidence to draw conclusions about the effects of beta-adrenoreceptor antagonists on perinatal outcome (Magee et al., 2007). The major concerns associated with these drugs are intrauterine growth retardation (IUGR), cardiorespiratory depression, bradycardia, hypoglycemia, and hypothermia (Ghanem & Movahed, 2008). Reports of these adverse effects are rare, and they are most often associated with atenolol (Butters, Kennedy, & Rubin, 1990).

Given the risk of IUGR associated with prolonged beta-blocker use, it is recommended that fetal growth be routinely monitored by ultrasound and that the mother’s hemodynamic status be closely observed (Howlett et al., 2010). There are thoughts that lower maternal blood pressures may increase the risk for developing IUGR; titration of these medications should be done slowly over time. Beta-blockers should be used very cautiously and are best avoided in cases of acutely decompensated heart failure in pregnant patients.

## Loop Diuretics

Loop diuretics play an important role in the management of the edema and pulmonary congestion associated with heart failure, and are thought to be safe for use in pregnancy (Briggs, Freeman, & Yaffe, 2008). These drugs work by inhibiting the reabsorption of sodium and chloride in the ascending loop of Henle and in the distal renal tubule, leading to an increased excretion of water, sodium, chloride, magnesium, and calcium (Brunton, Lazo, & Parker, 2005). The overall net effect causes an increase in diuresis and a decrease in cardiac preload.

While believed to be nonteratogenic, the risks and benefits of using these drugs throughout pregnancy must be considered. For example, diuretics have been shown to decrease placental perfusion and intravascular volume contraction (Carr, Gavrila, Brateng, & Easterling, 2007; Ghanem & Movahed, 2008; Newsstead-Angel & Gibson, 2009). Sibai, Grossman, & Grossman followed 21 patients in their first trimester currently taking diuretics prior to study enrollment. In order to compare outcomes, 10 patients were asked to discontinue treatment with their diuretic following enrollment. The findings showed that while initial plasma volumes were similar in the two groups, measurements at various stages of pregnancy showed decreased plasma volumes in the diuretic-treated group. However, there was no difference in perinatal outcomes between the two groups.

## Aldosterone Antagonists

Aldosterone antagonists, such as spironolactone and eplerenone, compete with aldosterone for receptor sites in the distal renal tubules, increasing sodium chloride and water excretion while retaining potassium and hydrogen ions (Brunton, Lazo, & Parker, 2005). These drugs are beneficial in patients with congestive heart failure and prolong survival in patients with NYHA class III and IV heart failure (The RALES Investigators, 1996; Pitt et al., 1999). There are currently no data to support the use of aldosterone antagonists during pregnancy, and animal studies have reported feminization of the male fetus due to the antiandrogen effects of the drugs (Briggs, Freeman, & Yaffe, 2008; Newsstead-Angel & Gibson, 2009). There is one case report of a patient with Bartter’s syndrome who was treated with spironolactone 200 mg/day during three pregnancies, of which two boys were reported to have developed mild learning disabilities (Groves & Corenblum, 1995). Until more safety data have been established, it is recommended that aldosterone antagonists be avoided during pregnancy (Briggs, Freeman, & Yaffe, 2008; Newsstead-Angel & Gibson, 2009).

## Hydralazine and Nitrates

Hydralazine is a centrally acting vasodilator. It causes direct vasodilation of arterioles and a decrease in systemic vascular resistance. When used in conjunction with nitrates, which are predominantly venodilators, it has been shown to provide symptomatic and mortality benefits particularly in certain populations of heart failure patients, such as African Americans (Taylor et al., 2004; Taylor et al., 2007). Both of these drugs have a record for safe and effective use in pregnancy without any evidence of teratogenicity and may serve as a good substitute for ACE inhibitors or ARBs in this patient population (Newstead-Angel & Gibson, 2009).

## Digoxin

Digoxin has not been shown to improve mortality in the general population of heart failure patients; as a result, it is not a first-line agent in the management of heart failure (The Digitalis Investigation Group, 1997). It is instead used as adjunct therapy to improve exercise tolerance and increase cardiac contractility. It works via inhibition of the sodium/potassium ATPase pump in myocardial cells, causing an influx of calcium via the sodium-calcium exchange pump that ultimately leads to an increase in cardiac contractility (Briggs, Freeman, & Yaffe, 2008).

Digoxin has historically been used in pregnant patients with heart failure as well as for the management of both maternal and fetal arrhythmias; it has been proven safe at all stages of pregnancy (Briggs, Freeman, & Yaffe., 2008). Some concerns, such as low birth weight and mental retardation, have arisen from anecdotal case reports (Widerhorn, Rubin, Frishman, & Elkayam, 1987), but overall, digoxin has been used with favorable results. Because of these pharmacokinetic alterations, serum levels may be checked periodically during the course of therapy in order to minimize the risk of toxicity while trying to achieve a therapeutic response (Widerhorn et al., 1987). This drug should be considered in pregnant women with heart failure who are still symptomatic despite adequate treatment with vasodilators and diuretic therapy.

## Management During Delivery

The decision regarding timing and mode of delivery for these high-risk patients is generally based on obstetric indications. Early delivery is not required unless medical management is unsuccessful and the patient is hemodynamically unstable (Howlett et al., 2010). For most cardiac conditions, a normal vaginal delivery is the preferred mode of delivery for the mother, as it is associated with minimal blood loss, greater hemodynamic stability, avoidance of surgical stress, and less chance of postoperative infection and pulmonary complications than cesarean section (Thorne, 2004). Effective pain management is necessary to avoid tachycardia, which increases myocardial oxygen consumption. Cesarean delivery is reserved for indications such as fetal distress or failure to progress (Howlett et al., 2010).

During delivery, maintenance of normal to low heart rate to decrease oxygen demand and prevention of large swings in blood pressure are imperative. Careful hemodynamic monitoring and fluid balance is obligatory and arterial and central venous pressure lines are recommended if cesarean section is chosen as a mode of delivery. Management in the intensive care unit is usually required due to the severity of the condition (Carlin & Alfirevic, 2010). Early critical care referral is essential for unstable and critically ill patients with pulmonary edema, hypoxia, mental obtundation, hypotension, refractory oliguria, or acidemia and may require Swan-Ganz monitoring, artificial ventilation, and inotropic support (Baughman, 2001).

## Postpartum Period

Subsequent monitoring after delivery depends on response to treatment, and includes a follow-up echocardiogram in the first several weeks to evaluate left ventricular systolic function. If standard heart failure medical therapy is ineffective, more aggressive ventricular support such as the intra-aortic balloon counter pulsation or left ventricular assist device may be considered (Carlin & Alfirevic, 2010). In the absence of evidence-based guidelines for the management of cancer patients with heart failure, standard heart failure therapy recommended by clinical guidelines including diuretics, beta-blockers, ACE inhibitors, nitrates, hydralazine, and digoxin should be initiated (Carlin & Alfirevic, 2010). Careful attention must be paid to fetal safety and to excretion of drug (i.e., ACE inhibitors and beta-blockers) or drug metabolites during breastfeeding after delivery.

## Implications for Advanced Practice

With a growing number of cancer survivors at a childbearing age, advanced practitioners will increasingly come across cancer patients who are pregnant or contemplating pregnancy. These patients may present with many challenges that require a personalized approach to the management of the mother and the fetus. Health-care providers must educate themselves about the early signs and symptoms of worsening heart failure so that the treatment is initiated at an early stage. Advanced practitioners are often the patient’s main source of information within the health-care system; therefore, they need to be able to assist patients or refer them to appropriate services. Advanced practitioners should also evaluate patients’ psychosocial needs and encourage them to seek professional help when necessary (Rimes, Gano, & Milbourne, 2008). Working as a multidisciplinary team will help achieve the best possible outcome for mothers and their babies.

## Conclusions

Pregnancy in cancer patients with preexisting heart disease should be managed by a multidisciplinary team of cardiologists, oncologists, obstetrician, perinatologists, anesthesiologists, advanced practitioners, nurses, and pharmacists. A collaborative effort is paramount during various stages of pregnancy and perinatal care; a successful outcome is possible, as documented in our case. It is important to realize that the treatment recommendations of pregnant patients with cancer will always rely on limited evidence. Each clinical situation is unique and requires a multidisciplinary approach. A registry will help establish guidelines for optimal management of pregnancy in cancer patients with heart failure.
